# Reproductive toxicity of combined effects of endocrine disruptors on human reproduction

**DOI:** 10.3389/fcell.2023.1162015

**Published:** 2023-05-12

**Authors:** Sulagna Dutta, Pallav Sengupta, Sovan Bagchi, Bhupender S. Chhikara, Aleš Pavlík, Petr Sláma, Shubhadeep Roychoudhury

**Affiliations:** ^1^ School of Medical Sciences, Bharath Institute of Higher Education and Research (BIHER), Chennai, Tamil Nadu, India; ^2^ Department of Biomedical Sciences, College of Medicine, Gulf Medical University, Ajman, United Arab Emirates; ^3^ Molecular Medicinal and Material NanoChemistry Laboratory, Department of Chemistry, Aditi Mahavidyalaya, University of Delhi, Delhi, India; ^4^ Laboratory of Animal Physiology, Department of Animal Morphology, Physiology and Genetics, Faculty of AgriSciences, Mendel University in Brno, Brno, Czechia; ^5^ Laboratory of Animal Immunology and Biotechnology, Department of Animal Morphology, Physiology and Genetics, Faculty of AgriSciences, Mendel University in Brno, Brno, Czechia; ^6^ Department of Life Science and Bioinformatics, Assam University, Silchar, India

**Keywords:** endocrine disrupting chemicals, metals, human fertility, infertility, oxidative stress, reductive stress

## Abstract

Confluence of environmental, genetic, and lifestyle variables is responsible for deterioration of human fecundity. Endocrine disruptors or endocrine disrupting chemicals (EDCs) may be found in a variety of foods, water, air, beverages, and tobacco smoke. It has been demonstrated in experimental investigations that a wide range of endocrine disrupting chemicals have negative effects on human reproductive function. However, evidence on the reproductive consequences of human exposure to endocrine disrupting chemicals is sparse and/or conflicting in the scientific literature. The combined toxicological assessment is a practical method for assessing the hazards of cocktails of chemicals, co-existing in the environment. The current review provides a comprehensive overview of studies emphasizing the combined toxicity of endocrine disrupting chemicals on human reproduction. Endocrine disrupting chemicals interact with each other to disrupt the different endocrine axes, resulting in severe gonadal dysfunctions. Transgenerational epigenetic effects have also been induced in germ cells, mostly through DNA methylation and epimutations. Similarly, after acute or chronic exposure to endocrine disrupting chemicals combinations, increased oxidative stress (OS), elevated antioxidant enzymatic activity, disrupted reproductive cycle, and reduced steroidogenesis are often reported consequences. The article also discusses the concentration addition (CA) and independent action (IA) prediction models, which reveal the importance of various synergistic actions of endocrine disrupting chemicals mixtures. More crucially, this evidence-based study addresses the research limitations and information gaps, as well as particularly presents the future research views on combined endocrine disrupting chemicals toxicity on human reproduction.

## 1 Introduction

According to the US Environment Protection Agency, an endocrine-disrupting chemical (EDC) refers to an external substance that disrupts the body’s natural hormone production, release, transportation, metabolism, binding, action, or removal processes. These hormones play a crucial role in maintaining homeostasis and regulating developmental processes (Kavlock et al., 1996). Several notable EDCs include antibiotics such as amoxicillin, penicillin, sulfonamides, and tetracycline, antidepressants such as citalopram and fluoxetine, non-steroidal anti-inflammatory drugs (NSAIDs) like naproxen, diclofenac, ibuprofen, contraceptive pills, various household and industrial chemicals such as alkyl phenol detergents, dioxins, fire retardants, polychlorinated biphenyls (PCBs), phthalates, and phthalate esters (PAEs) (Christensen et al., 2006; Caliman and Gavrilescu, 2009; Pal et al., 2010; Fiorentino et al., 2017).

Research undertaken in the domain of EDCs has substantially expanded over the last two decades, owing to the potential harm that they can cause to the human body as well as advances in the fields of environmental toxicology and developmental biology. While there is ongoing debate regarding the hypothesis that human reproductive functions are adversely affected by exposure to EDCs, numerous studies have depicted a notable reduction in fertility biomarkers, particularly sperm counts, in human subjects exposed to these substances (Axelstad et al., 2018; Cannarella et al., 2022). It is widely acknowledged that EDCs can have detrimental effects on human reproduction (Andersson et al., 2008; Safe, 2013). EDCs-induced reproductive dysfunctions are mainly through estrogen- or androgen-mediated mechanisms (Jouannet et al., 2001; Perry et al., 2007; Safe, 2013).

The harmful consequences of EDCs have resulted in their usage being restricted in nations where there is adequate confirmation on wide exposure to these chemicals (Knez, 2013). Some EDCs, such as polybrominated diphenyl ethers and PCBs, have been outlawed in various westernized countries, including the United Kingdom. However, in other circumstances, when these types of EDCs are used in occupation or contaminate the environment, human exposure to such substances is unavoidable. Pesticides, for example, are a chemical class that can be used as an example. The term “pesticide” refers to “any substance or combination of substances, that is used to prevent or eradicate unwanted insects, including vectors of disease in humans and animals, weeds, or fungi, in order to enhance food production and aid in the processing, storage, transportation, or marketing of food and agricultural commodities” (Sifakis et al., 2011; Mehrpour et al., 2014). Pesticides can be EDCs based on their structure and principle of action adopted. Organochlorines and organophosphorus pesticides are examples of insecticides having endocrine disrupting effects.

When organisms are treated with EDC combinations, large levels can bioaccumulate, causing alterations in androgen synthesis and catabolism, impaired fertility, and multiple severe problems (Christensen et al., 2006; Kabir et al., 2015; Zhou et al., 2017). Presently, there is a surge in the quantity of investigations conducted to examine the effects of combinations of chemicals on the endocrine system (Ding et al., 2017). Integrating dose-response assessment through *in vitro*/*vivo* experimentation and *in silico* mathematical modeling provides a comprehensive strategy for toxicological evaluation, which is essential for prioritizing hazardous chemical mixtures in the pursuit of safer chemical research. Toxicological concerns associated with chemical mixtures are often underestimated by standard risk assessment tools (Barata et al., 2006; Dietrich et al., 2010). However, this method has the potential to understate toxicological consequences, particularly for medicines with several modes of action (MOAs) (Brosche and Backhaus, 2010; Ding et al., 2017). Classical null hypothesis models predict molecular interactions at target sites and undesired combined effects. Some of these models are concentration addition (CA), independent addition (IA), and combination index (CI).

The present article comprehensively reviews the influence of EDCs on human reproductive functions, various modes of their actions and the toxicokinetics of cocktail EDCs mixtures, to aid further much needed research in this field for greater insights into the combined effects of EDCs on human reproduction.

Literature search was conducted using electronic databases using the string, (“endocrine disrupt*” OR “edc”) AND (“sperm*” OR “male reproduct*” OR “male fertility” OR “female reproduct*” OR “female fertility” OR “polycystic ovarian syndrome” OR “pcos”). Relevant full-length articles limited to English language and human studies were reviewed for relevant information. As the topic comprises a vast scope and is being research interest since more than last three decades till the present day, the literature search could not be restricted by years of publications. This is the reason why it was decided to write a comprehensive literature review rather than a systematic review on the influence of EDCs on human reproduction.

## 2 Sources of EDCs

EDCs have a variety of physicochemical properties which degrade and react differently in environment, influencing the exposure routes for humans and animals (Karthikeyan et al., 2019). Higher sunlight, temperature increase, and aerobic conditions are the key abiotic elements that boost degradation processes, hence, warmer regions of the world aid faster degradation. It is possible that in the environment, other chemical transformations (such as oxidation, hydrolysis, and photochemical reactions) will take place. Some putative EDCs that do not break down fast in the environment, such as persistent organic pollutants (POPs), may accumulate in certain compartments (such as sediment) or be transported to vast distances from their original sources (Sifakis et al., 2011; Sifakis et al., 2017; Annamalai and Namasivayam, 2015).

Air, water, soil, and food can all be possible sources EDCs exposure for human (Pi et al., 2017). By ingesting, inhaling, or coming into touch with the skin, the chemicals can pass through cell membranes and enter the body (Tchounwou et al., 2012; Annamalai and Namasivayam, 2015).

### 2.1 Exposure through food

A frequent route of human exposure to EDCs is via food intake, which can lead to bioaccumulation and biomagnification (Muncke, 2011). Dietary exposure has varying effects based on dietary choices, food chain location, species and quantity ingested. Persistent and lipophilic organic pollutants typically bioaccumulate in species placed at the top of food chain. POPs concentrations in fish-eating birds and marine animals are several times higher than in the fish they eat or in the surrounding seas. The levels can be multiplied by hundreds of millions (World Health Organization, 2002; Muncke, 2011).

Humans eat both animal and plant derived foods, and as dietary preferences differ by population, culture, and geographical region. EDCs exposure also varies greatly among individuals (Muncke, 2011). POPs levels have been found to be greater in humans who rely on contaminated animals for sustenance (Li et al., 2006). People in Greenland and Canada, as well as women of Faroe Islands, have been found to have some of the highest POPs concentrations (Park et al., 2007; Long et al., 2015). In contrary, people who consume large quantities of toxic fish have been found to have greater levels of persistent lipophilic substances in their bodies (Dorea, 2006). Breast milk has the potential to be a significant source of EDC exposure for newborns, especially during a critical period of growth and development (Stefanidou et al., 2009). EDC levels in bottled baby milk and infant meals are poorly understood.

The effects of persistent lipophilic substances on human health have been investigated extensively. The European Union has published a thorough study of food intake of polychlorinated dibenzodioxins and dibenzofurans in ten nations (EU-SCOOP, 2000). According to reports, dairy products, fish and fish products, meat, and meat products are the primary sources of exposure to endocrine-disrupting chemicals (EDCs) in developed countries. The proportions of EDC exposure may vary depending on the food source or exposure scenario (EU-SCOOP, 2000; Muncke, 2011).

### 2.2 Exposure through ambient air

The intrinsic and relative volatility of every chemical in contact with water, vegetation, and soil determines its air concentration. When estimating exposure regionally or globally, meteorological factors (such as wind speed, temperature, humidity) may impact upon the contaminant concentration in the ambient air (Annamalai and Namasivayam, 2015). In particulate matter in the air, less volatile compounds may also be present. Semi-volatile compounds, which make up the bulk of known EDCs, can bind to airborne particles, affecting absorption into the circulation and gastric uptake (Rudel et al., 2010; Salgueiro-Gonzalez et al., 2015). Other EDCs in the air can be deposited on soil, leaves, and grass in terrestrial systems, as well as in aquatic systems (Woodrow et al., 2019). Experimental data or model estimations are needed to calculate the absorbed dosage of an EDC from air. When the air concentrations, breathing rate, and absorption efficiency are known, it is possible to estimate or compute the inhaled dose (Little et al., 2012).

### 2.3 Exposure via water

Water is both the living environment for many aquatic species (e.g., fish, aquatic invertebrates) and used for drinking and other purposes by the humans and terrestrial species. Surface waters have been shown to include various industrial pollutants, pesticides, and natural hormones. Chemicals can be dissolved in water and/or attached to particles (Geyer et al., 2000).

Humans may consume EDCs with drinking water however, it is not a common route unless there has been significant pollution. Microbial contaminants, suspended particulate matter, and some toxic compounds are all removed from drinking water in affluent countries (e.g., pesticides, aromatic hydrocarbons) (Wee and Aris, 2017). In certain circumstances, water treatment may include other chemical contaminants. Drinking water in underdeveloped countries is rarely treated, and it is frequently contaminated with industrial and naturally occurring pollutants. The absorbed dosage of a pollutant from water can be calculated using models (Wee and Aris, 2017). Drinking water is not thought to be a major source of persistent lipophilic chemical exposure (Yilmaz et al., 2020).

### 2.4 Exposure through soil

Various EDCs (e.g., PCBs, dioxins, and polybrominated diphenyl ethers—PBDEs) have been found in soils and/or sewage effluent from throughout the world (Ying and Kookana, 2005; Sangeetha et al., 2021). This might be a major source of exposure to EDCs for species that live near the soil. Some birds and terrestrial animals eat these organisms (such as snails, insects, and worms). Grazing exposes farm animals to polluted soil, exposing humans via the food chain (Yang et al., 2006).

### 2.5 Occupational exposure to EDCs

Organophosphates (OPs) are among the most extensively used pesticides in agriculture (Koureas et al., 2014). Paraoxonase (PON) enzyme and Cytochrome P450 (CYP) families are xenobiotic metabolizing enzymes that process these EDCs (Androutsopoulos et al., 2013). The presence of secondary metabolites such as dialkyl phosphates in biofluids indicates exposure to Ops (Androutsopoulos et al., 2013; Sokoloff et al., 2016). Exposure of humans to organophosphates (OPs) can manifest through diverse pathways, including environmental or occupational routes. The extent of occupational exposure to these chemicals can be contingent upon a multitude of factors, such as the utilization of personal protective equipment (PPE) encompassing gloves, boots, and glasses, the implementation of precautionary measures like wearing protective clothing and frequent hand washing, the nature of the profession, the accessibility of specialized training for safe handling and usage of pesticides, and the presence of regulatory measures in the form of directives and laws directed at safeguarding workers from exposure to occupational toxins, as well as the undertaking of periodic health monitoring (Mehrpour et al., 2014). Dermal exposure of EDCs often occurs from the plant surface to the farmer’s skin (Kapka-Skrzypczak et al., 2011). However, exposure can occur even when wearing protective clothes, as this might be a source of extra exposure (Poet and McDougal, 2002). Plasma cholinesterase activity has been used to assess chronic OP exposure (De Silva et al., 2006).

## 3 EDCs and human reproductive functions

### 3.1 Impact of EDCs on female reproduction

The female reproductive system can experience negative consequences caused by exposure to different EDCs. These stem from their impacts on folliculogenesis. Primary follicles and antral follicles develop from primordial follicles. Bisphenol A (BPA), 2,3,7,8-tetrachlorodibenzo-p-dioxin (TCDD), methoxychlor (MTX), and phthalates are examples of EDCs that can interfere with the formation of the ovarian follicles. EDCs toxicity to antral follicles can cause infertility. EDCs have been related to female reproductive disorders, endometriosis and PCOS, as well as decreased oocytes number and antral follicle in women undertaking fertility treatments (Souter et al., 2013; Caserta et al., 2014). Experimental animal research has confirmed the findings in human, showing that exposure to EDCs reduces the primordial follicle-pool and causes early ovarian failure (Wang et al., 2014). Some EDCs, such as MTX, can inhibit the development of follicles by activating the expression of anti-Müllerian hormone (AMH) in the fluid-filled sacs containing immature eggs (antral follicles), while TCDD can impede the production of ovarian steroids and hinder cell growth in the ovaries of rodents (Uzumcu et al., 2006). Following 60 days of exposure to phthalate (DHEP) at 600 mg/kg, rats showed increased atretic follicles as well as a reduced number of main and secondary follicles (Xu et al., 2010) (Table 1).

**TABLE 1:** Contemporary human studies on the impact of endocrine disrupting chemicals (EDCs) on reproductive functions.

References	EDCs	Findings
Zhu et al. (2022)	Triclosan (2,4,4′-trichloro-2′-hydroxy-diphenyl ether, TCS)	The association between TCS exposure and male fecundity was investigated in a cohort of couples in Shanghai, China, where elevated TCS concentrations in male urine were found to be associated with diminished fecundability and an increased risk of infertility
Holmboe et al. (2022)	Bisphenol A (BPA), benzophenone-3 (BP-3)	High exposure to bisphenol A (BPA) and benzophenone-3 (BP-3) is associated with compensated reduced Leydig cell function, as observed by increased luteinizing hormone (LH) levels, but no other changes in markers of reproductive health
Maggio et al. (2022)	Organochlorine 1,1-dichloro-2,2-bis(p-chlorophenyl)ethylene (DDE)	This study examined the effects of environmental exposures to EDCs on the sperm methylome of a cohort of men from the Faroe Islands. Results showed that exposure to the organochlorine DDE was associated with genome-wide differentially methylated regions (DMRs) in genes involved in neurological functions and neurodevelopmental processes frequently impacted by autism spectrum disorder (ASD), suggesting a possible mechanism for transgenerational inheritance of ASD and other disorders due to altered methylation of ASD risk genes
Berghuis et al. (2022)	Polychlorinated biphenyls (PCBs)	The study investigated the association between prenatal exposure to persistent organic pollutants (POPs), including PCBs, and pubertal development in 13- to 15-year-old children. The findings suggest that higher prenatal PCB exposure could lead to more advanced pubertal development in both boys and girls, as indicated by higher levels of testosterone, pubic hair, breast development, and larger testicular volume
Lecante et al. (2022)	Acetaminophen (APAP)	The study investigated the effects of APAP on fetal human ovaries in culture and found that it behaves as an endocrine disruptor in the fetal human ovary by reducing cell number, inducing cell death, and decreasing KI67-positive cell density, which can negatively impact first-trimester human fetal ovarian development, especially during a 10- to 12-DW window of heightened sensitivity. Additionally, APAP targeted subpopulations of germ cells and disrupted human fetal ovarian steroidogenesis, without affecting prostaglandin or inhibin B production, suggesting a potential risk to women’s reproductive health from *in utero* exposure to APAP.
Lavogina et al. (2022)	Dichlorodiphenyldichloroethylene (p,p'-DDE), hexachlorobenzene (HCB) and perfluorooctanesulfonic acid (PFOS)	This study investigated the effect of nine selected EDCs on primary human endometrial stromal cell decidualization *in vitro*, and found that p,p’-DDE, HCB, and PFOS significantly reduced decidualization, indicating that EDCs commonly present in the blood circulation of reproductive-aged women can reduce decidualization of human endometrial stromal cells *in vitro*, which could have implications for endometrial dysfunction and implantation failure in women
Barnett-Itzhaki et al. (2021)	Phthalate	This prospective study examined the association between phthalate metabolite concentrations in follicular fluid (FF) and extracellular vesicle microRNA (EV-miRNA) expression profiles in FF from 105 women. Results showed that phthalate metabolite concentrations were associated with the expression of EV-miRNAs and their associated pathways, which may be involved in ovary or oocyte development, maturation, and fertilization, suggesting a possible mechanism by which endocrine disruptor chemicals may interfere with female fertility
Park and Chung (2021)	Bisphenol A (BPA)	This study aimed to investigate the effects of a dietary modification intervention on menstrual pain and urinary BPA levels in female college students. The intervention was effective in reducing menstrual pain and urinary BPA levels, with higher adherence associated with greater reduction in menstrual pain, indicating the potential for dietary modification to mitigate the adverse effects of EDCs
Zhang S et al. (2021)	Ten commonly exposed EDCs including urinary phthalate metabolites, equol, and whole blood heavy metals (mono-n-butyl-, monoethyl-, mono-2-ethylhexyl-, mono-benzyl-, Mono(3-carboxypropyl)-, mono-isobutyl- phthalate, cadmium, lead, mercury and equol)	This study has assessed the combined effects of commonly exposed EDCs on uterine leiomyomata (UL) and endometriosis (EM) using multivariable logistic regression model, weighted quantile sum (WQS) regression, and Bayesian kernel machine regression (BKMR) models. Equol and mercury were positively associated with UL, while the WQS index had a marginally positive association with EM, particularly in premenopausal women, indicating a need for further research on the associations between these chemicals and reproductive health outcomes
Syrkasheva et al. (2021)	Bisphenol A (BPA)	This study investigated the association between the levels of BPA in blood and follicular fluid, polymorphism of detoxification system genes, and *in vitro* fertilization (IVF) outcomes. BPA was detected in the majority of blood samples but less frequently in follicular fluid, and there was no correlation between BPA levels in the two fluids. The study also found that the absence of the A allele of the SULT1A1 gene was associated with a higher frequency of BPA detection in follicular fluid, suggesting the role of detoxification system genes in BPA metabolism. However, further research is needed to determine the impact of BPA and detoxification system genes on IVF outcomes

Although EDCs reportedly have several deleterious effects on female reproductive functions in experimental animals (Table 2), in human, impacts of EDCs on female fertility need further studies (Ehrlich et al., 2012; Philippat et al., 2012; Rochester, 2013). The seeming contradiction on effect of EDCs on human female reproduction is based on the duration and intensity of exposure, which determines the daily exposure to EDCs. In contrast, in laboratory studies it is easier to determine daily doses of EDCs where circumstances and most importantly the EDCs induction protocols are adequately controlled. The differences in how EDCs affect living organisms in various studies are caused by differences in experimental conditions and methods used to determine the exposure dose. Although EDCs’ action mechanism can be shown in animal models, the dose used is often not equivalent to real human exposure. For example, DEHP treatment from day 14 to day 19 of gestation caused mineralocorticoid receptor dysfunction in rats (Martinez-Arguelles et al., 2009). The animals were given a much higher dose of MTX than typical human DEHP exposure, but since they were at an early stage of development similar to human infancy, the harmful effects were reduced. Uzumcu et al. (2006) used concentrations of 1–500 mg/kg of MTX in female rats after birth and found that ovarian weight decreased with treatment at 50, 100, and 500 mg/kg. Unlike the previous investigations, in their study, Wang et al. (2014) administered doses of 0.5, 20, and 50 g/kg of BPA to newborn FVB mice in order to demonstrate a reduction in fertility caused by BPA. While the doses used in this study were similar to levels of BPA exposure experienced by humans, the majority of human studies have focused on adult individuals rather than neonatal ones, with exposure levels of 2.78 ng/mL in urine and 1.6 g/L in serum. The study’s results indicate that both the experimental dose of BPA exposure and the target population selected were significant factors in identifying the negative impacts of EDCs. Additionally, research on humans and EDCs is less common than animal and *in vitro* studies (Rochester, 2013).

**TABLE 2:** Contemporary animal studies on the impact of endocrine disrupting chemicals (EDCs) on reproductive functions.

References	EDCs	Findings
Li et al. (2022)	Di-(2-ethylhexyl) phthalate (DEHP) and polystyrene nanoparticles (PSNPs)	This study aimed to assess the effects of coexposure to DEHP and PSNPs on the male reproductive system. Results showed that DEHP and PSNP cotreatment induced more severe impairment on sperm quality, testicular and epididymal histology, and gene expression than DEHP treatment alone. Transcriptomic analysis revealed differential regulation of genes involved in immune response, signaling pathways, oxidative stress, and ATP synthesis. These findings emphasize the potential for environmental pollutants to disrupt male reproductive function and the potential for nanoparticles to exacerbate their toxic effects
Yun et al. (2022)	Atrazine	The study aimed to determine if exposure to low levels of atrazine affects oocyte chromosome segregation and exacerbates aging-related aneuploidy in female mice. The study found that exposure to atrazine during development or adulthood caused persistent changes to the female germline, leading to lower oocyte quality, chromosomal abnormalities, and reduced fertility, potentially impacting reproductive lifespan and congenital disease
Shi et al. (2022)	2,2′,4,4′-tetrabromodiphenyl ether (BDE-47)	Female zebrafish were exposed to different concentrations of BDE-47 for 21 days. Results showed that high levels of BDE-47 caused impaired oocyte development and structure, decreased sex hormone levels, induced oxidative damage, and altered the expression of genes related to the hypothalamic pituitary-gonad axis
Desmarchais et al. (2022)	Bisphenol S (BPS)	This study examined the effects of chronic exposure to BPS on folliculogenesis and embryo production in adult ewes, and how it is influenced by their metabolic status. The results suggest that while BPS exposure did not affect follicular population or oocyte competence, it did have a significant interaction with diet on embryo production, indicating that BPS effects are modulated by metabolic status. Further research is needed to determine the potential reproductive health risks of BPS exposure
Yao et al. (2022)	Cadmium	This study investigated the potential protective effect of letrozole, an aromatase inhibitor that stimulates spermatogenesis, on cadmium-induced reproductive toxicity in male mice. Cadmium exposure caused significant decreases in body weight, sperm count, motility, vitality, and plasma testosterone levels, while letrozole treatment compensated for deficits in sperm parameters induced by cadmium and increased serum testosterone levels. RNA-seq analysis showed that letrozole treatment affected steroid biosynthetic processes and upregulated testosterone synthesis-related genes, which might mediate the protective effect of letrozole on cadmium-induced reproductive toxicity
Zhang et al. (2022)	Di-(2-ethylhexyl) phthalate (DEHP)	This study used network pharmacology and animal models to investigate the potential targets and mechanisms of a traditional Chinese medicine, WFY, in repairing sperm DNA damage caused by DEHP. Results suggest that WFY may act through the PI3K/Akt and metabolic pathways, and may target Bhmt to repair sperm DNA damage. These findings offer a potential new approach for using traditional Chinese medicine to prevent and treat reproductive system injury caused by pollutants
Téteau et al. (2022)	Bisphenol S (BPS)	The study aimed to examine the effect of chronic exposure to BPS on steroid hormones in the ovary, oviduct and plasma using ewes as a model, and the effect was tested based on two diet groups. The results showed that BPS acted as an endocrine disruptor in ewes with differential actions according to metabolic status, and exposure to BPS impaired estradiol concentrations in both follicular and oviduct fluids of well-fed ewes and progesterone, estradiol, and estrone concentrations in plasma of restricted ewes
Guo et al. (2022)	Polycyclic aromatic hydrocarbons (PAHs)	Low-level exposure to phenanthrene, a type of low-molecular-weight PAHs, during gestation in mice led to reproductive disorders in F1 adult female mice. The treatment increased the number of immature follicles, inhibited oocyte maturation, reduced hormone levels, and upregulated certain receptors and pathways in the ovary, resulting in exacerbated follicular atresia. The study suggests that the reproductive toxicity of low-molecular-weight PAHs cannot be ignored
Zhang Y et al. (2021)	4,4′-(9-Fluorenylidene) diphenol (BPFL)	The study investigated the effects of BPFL on porcine Sertoli cells (SCs) and the protective role of chlorogenic acid (CA) against BPFL-induced impairments. BPFL exposure caused cell damage, oxidative stress, mitochondrial dysfunction, DNA damage, endoplasmic reticulum stress, and apoptosis in a dose-dependent manner. However, CA supplementation alleviated the harmful effects of BPFL on porcine SCs
López-Rodríguez et al. (2021)	Di-n-butyl phthalate, di(2-ethylhexyl) phthalate, vinclozolin, prochloraz, procymidone, linuron, epoxiconazole, dichlorodiphenyldichloroethylene, 4-methyl-benzylidene camphor, butylparaben, bisphenol A, acetaminophen	Environmentally relevant dose of EDC mixture given to rats during gestation and lactation resulted in multi- and transgenerational disruptions of sexual maturation and maternal care via epigenetic reprogramming of the hypothalamus. Female rats of germ cell (F2) and transgenerational exposure (F3) had delayed pubertal onset and altered folliculogenesis, while hypothalamic polycomb group of epigenetic repressors played a vital role in transgenerational transmission of disrupted reproductive phenotypes. Additionally, there was a multigenerational reduction of maternal behavior, which was linked to a loss of hypothalamic dopaminergic signaling. This study highlights the possible impact of EDC mixtures on future generations

Having increased exposure to BPA was linked with worse ovarian function, including a decreased count in mature and fertilized eggs, and also a lower peak level of the hormone E2 in response to hCG stimulation. In an occupational case-control analysis of 155 female participants, a positive correlation was discovered between exposure to BPA and elevated levels of the sex hormone prolactin relative to unexposed subjects. Similarly, a cross-sectional study consisting of 171 women indicated that BPA exposure was linked to the incidence of polycystic ovary syndrome (PCOS) (Kandaraki et al., 2011).

Studies by Yang et al. (2009) and Aschengrau et al. (1998) found no significant links between BPA and breast cancer development, while Sugiura-Ogasawara et al. (2005) associated it to abnormal neonatal development, early birth, and miscarriages. The results of the above investigations show that BPA is extremely harmful to female fertility. Except for the study by Aschengrau et al. (1998), most of other studies had lesser sample sizes than 200. This means adequate future studies are needed to reach consensus. On the basis of outcomes such as embryo quality, beta–hCG, and reduced likelihood of conception, infertile couples and/or couples undergoing IVF showed to have higher susceptibility to BPA than other couples (Ehrlich et al., 2012; Chen L et al., 2013). A third of these women had undiagnosed infertility, while another third had unexplained infertility (Ehrlich et al., 2012; Chen M et al., 2013). Consequently, the susceptibility to BPA among the observed cohorts cannot be attributed solely to male or female infertility. Through an investigation conducted at 14 distinct clinical facilities in the United States, it was determined that specific phthalate metabolites, as opposed to BPA, exhibited a correlation with the detection of endometriosis (Louis et al., 2013; Caserta et al., 2014). Thus, the literature suggests that BPA may contribute to human infertility (Rochester, 2013).

Several other EDCs have been shown to have similar effects to BPA in humans. For example, epidemiological data have supported female reproductive issues and developmental exposure to organochlorine pesticides (Zama and Uzumcu, 2009; 2010). Even though phthalates are expansively investigated for their impact on male reproductive functions, evidence is mounting that they also impair female fertility. D-ethylhexyl phthalate and its metabolite, mono-2-ethylhexyl phthalate (MEHP) are the most investigated (Craig et al., 2011). The levels of phthalates have been linked to an increased risk of implantation failure in infertile patients (Hauser et al., 2016). The dearth of human data regarding DDT and dioxin-like compounds stems from their prohibition for more than three decades. Conversely, the levels of HCB exhibited a positive correlation with implantation malfunction, whereas no marked divergence was discerned between DDE and DDT (Mahalingaiah et al., 2012). Increasing evidence links OPs to disturbance of physiological functions of female reproductive system. Raanan and associates (Raanan et al., 2015) linked greater prenatal DAP concentrations to respiratory problems in children aged 0.5–5 years. A similar research conducted in China revealed that the primary risk factor for neurobehavioral development in newborns was elevated levels of DAPs and their metabolites detected in urine (Zhang et al., 2014). Regarding the association between exposure to OPs and female reproductive disorders and infertility, the preponderance of evidence in the scientific literature is derived from *in vivo* investigations. Specifically, investigations have linked the activity of PON1 enzyme in foetuses and early childhood to compromised foetal growth and development following prenatal exposure to OPs. Consequently, exposure to OPs elevates the vulnerability of foetuses and children to OP poisoning and may exert a greater impact on growth when compared to the effects of pesticide exposure in adults (Peiris-John and Wickremasinghe, 2008).

In summary, the literature suggests association between EDCs and female reproductive system disorders. In terms of the mechanism, aromatase inhibition by EDCs contributes to the development of defective oocytes, which is one of the main causes of infertility. There are many disparities among studies, including methodological complexity, occupational or environmental exposure, and sample size. The time of exposure is critical since the same dose has varied effects on subjects of different ages. Furthermore, exposure during important developmental phases (prenatal, neonatal, and childhood) is thought to influence the reproductive system more than adult exposure. Since exposure is defined by a mix of toxicants, the effects mentioned may be synergistic or antagonistic. The effects can occur immediately or later in life and generations, with varying sensitivity based on the genetic polymorphisms of the exposed individuals.

Innovative foci in disease investigation have emerged, encompassing obesity, type 2 diabetes, and hepatic steatosis, all intimately linked to developmental exposures to EDCs. Additionally, emphasis is placed on exploring novel EDCs, molecular pathways, devising advanced biomarkers for exposure and pathological outcomes, as well as investigations concentrating on interventional and preventative measures. Maternal exposure to environmental xenobiotics bears implications not solely for offspring health but also maternal wellbeing, signifying gestation as a susceptible period for environmental exposures (Heindel, 2019).

Two distinct methodologies have accentuated the significance of gestation as a vulnerable interval, an initial study observed altered mammary gland morphogenesis in dams following perfluorooctanoic acid (PFOA) exposure during pregnancy (White et al., 2007). It was demonstrated that BPA exposure throughout gestation in murine models resulted in diminished insulin secretion, perturbed glucose homeostasis, and augmented weight gain several months *postpartum*, indicating a predisposition to type 2 diabetes and obesity (Alonso-Magdalena et al., 2015). In a separate model, La Plante et al. revealed that pregnant mice subjected to bisphenol S (a BPA analogue) exposure until postnatal day 20 exhibited reduced lactation capabilities and altered nursing behavior, consequently impairing pup growth and maturation (LaPlante et al., 2017).

Concentrating on maternal health during pregnancy constitutes a novel dimension of vulnerability warranting consideration. It is imperative to elucidate the physiological alterations necessitated to establish a period of heightened sensitivity or susceptibility to disease, as well as the underlying mechanisms. A considerable research imperative is to contemplate the repercussions of multiple interactive exposure windows in the etiology of disease and dysfunction.

### 3.2 Impact of EDCs on male reproduction

**FIGURE 1:**
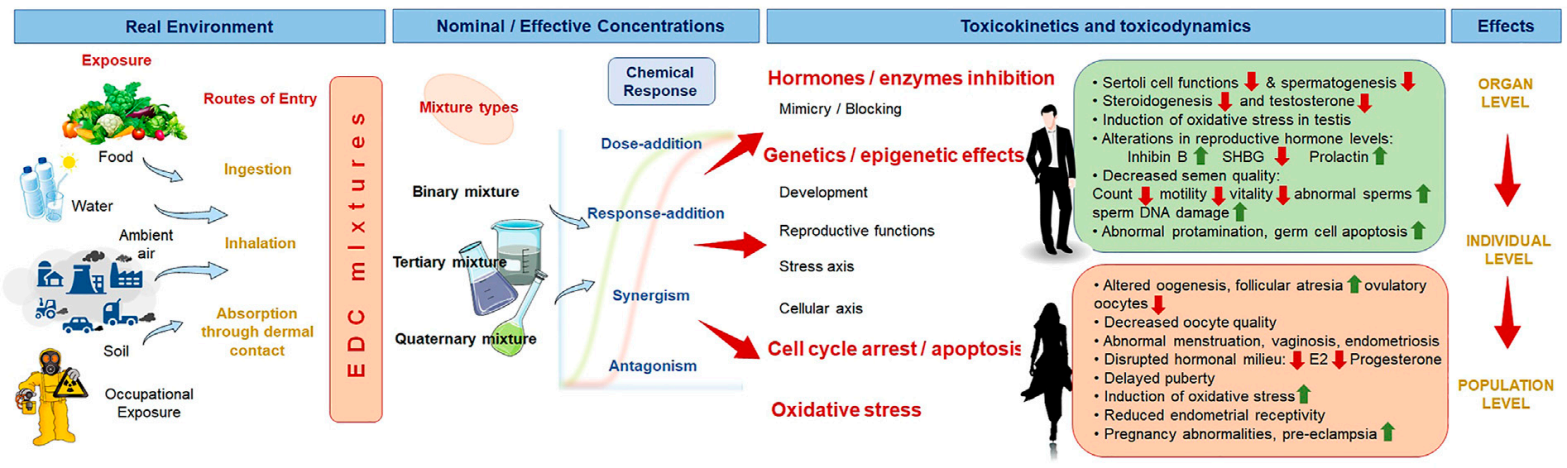
Reproductive dysfunctions by the combined effects of environment and lifestyle derived endocrine disrupting chemicals (EDCs). SHBG, sex hormone-binding globulin; E2, estradiol.

Over the past few decades, there has been growing apprehension regarding the adverse impact of Endocrine Disrupting Chemicals (EDCs) on the male reproductive system. Although several early 1940s studies suggested that EDCs-induced main adverse effect on the male fertility was decrease in semen quality, however, as a result of the disparate findings from diverse study designs, it was impossible to draw concrete conclusions regarding the dose, degree of exposure, and specific component responsible for the negative impacts. Cryptorchidism and testicular cancer are two examples of EDC-mediated male reproductive disorders that follow a similar mode as the effects of EDCs on female (Nordkap et al., 2012). These adverse effects, combined with a decline in semen quality, are key risk factors for EDC-mediated male fertility disorders (Figure 1).

The impact of EDCs on the male reproductive system arises from their modulation of steroid hormones, which stimulate prostate growth and promote the differentiation of the Wolffian ducts into the epididymis, vas deferens, and seminal vesicles. EDCs act as inhibitors of the enzymes 5α–reductase and aromatase, which are crucial for the biosynthesis of testosterone and estrogen via the conversion of androgens (Sweeney et al., 2015). Certain EDCs also function by modulating the expression of the two estrogen receptor isoforms, ERα and ERβ. Human ER expression is maintained during morphogenesis and cell differentiation but declines during early puberty. Increased plasma androgen levels may be linked to prostate cancer. Early BPA exposure improves estrogen–induced hyperplasia sensitivity in neonatal rats (Ho et al., 2006). This impact may be attributable to BPA’s androgenic action, however epigenetic etiologies for prostate cell proliferation genes have also been hypothesized (Ho et al., 2006; Sweeney et al., 2015) (Tables 1, 2).

#### 3.2.1 Effects of EDCs on semen quality

The assessment of sperm quality has emerged as a critical determinant of EDC-induced adverse outcomes. However, the early investigations conducted during the 1980s were merely observational and failed to address the impact of EDC exposure on the developmental anomalies of the male reproductive system. A series of studies were therefore carried out to comprehend the reasons behind the declining semen quality in various groups of men. One such study by Carlsen et al. (1992) highlighted a significant reduction in the average sperm concentration from 113 to 66 million/mL. Although several confounding factors including abstinence duration, semen analysis techniques, age, and fertility were taken into account during the analysis, the evidence was insufficient to establish a definitive conclusion (Jouannet et al., 2001). Also, geographic variance may have influenced this declining trend. With the confounding factors addressed, Swan and co-workers confirmed that the above-mentioned findings were not due to bias. They detected an annual reduction in sperm concentration of 1.5 and 3.1 percent among the population in the United States and Europe, respectively (Swan et al., 1997). Their meta-analysis confirmed that regional variance in sperm quality trends can be influenced (Swan et al., 2000). Furthermore, research from North Europe, particularly Denmark, have shown that the location of the human population studied can greatly influence semen quality (Jorgensen et al., 2001; Jorgensen et al., 2002). The lowest semen quality was found in Danish men, followed by Norwegian, Scottish, and French, with the highest quality found in Finnish males (Jorgensen et al., 2001; Jorgensen et al., 2002). Unlike Jorgensen and colleagues’ initial findings in 2001, subsequent investigations in Finland indicated a disheartening temporal decline in semen quality in men from the general population between 1998 and 2006 (Jorgensen et al., 2011; Jorgensen et al., 2012). Jorgensen and team’s research suggest that a considerable proportion of young males (about 43%) have poor sperm counts (less than 40 million/mL) (Andersson et al., 2008; Jorgensen et al., 2011; Jorgensen et al., 2012). According to the above epidemiological studies, the reduction in sperm count is not solely due to the geographical location. Other environmental considerations must be addressed for the higher occurrence of low semen quality in some North European groups.

Recent research on pesticide exposure and sperm quality has supported Jorgensen and colleagues’ findings. Pesticide exposure raises the chance of morphological abnormalities in sperm of agricultural workers, such as a decrease in sperm count and sperm viability. Pesticides like parathion and methyl parathion can damage the seminiferous epithelium, reducing sperm concentration and seminal volume (Yucra et al., 2006; Perry et al., 2007). Pesticide exposure diminishes seminal volume, raises pH, and alters sperm head morphology (Yucra et al., 2006). Lifeng et al. (2006) have reported that pyrethroid insecticides like fenvalerate can decrease sperm motility. Organochlorine pesticides like endosulfan and DDT have been linked to deleterious effects on the male reproductive system due to disturbance of the hypothalamic–pituitary–testicular axis and direct contact with sex steroid receptors in target tissue (Mehrpour et al., 2014). Male farmers from three separate communities in Malaysia were exposed to malathion and/or paraquat, resulting in reduced sperm concentration, pH, mean volumes, and motility of sperm cells (Hossain et al., 2010). In experiments on adult rats, malathion and parathion both reduced body weight, reproductive organ weight, and sperm counts (Narayana et al., 2006; Geng et al., 2015). There was a link between the stimulation of spermatogenic apoptosis by manipulation of Bax and Bcl-2 expression and a reduction in LH, FSH and testosterone levels (Geng et al., 2015). Indirect pesticide effect involves alteration of neuroendocrine control of testosterone production and release, excess generation of free radicals, and development of oxidative stress (Figa-Talamanca et al., 2001; Abdollahi et al., 2004). In 266 fertile men, polychlorinated biphenyls had a direct relationship with testosterone/estradiol hormonal concentration, demonstrating that structurally comparable chemicals to organochlorine pesticides with endocrine disruptive activities can impact the male reproductive system (Petersen et al., 2015). *Ex vivo* investigations confirmed the negative effects of phthalates on sperm quality by incubating spermatozoa with high concentrations of phthalates. Long-term (>3 days) exposure of human semen samples to the metabolite DEHP reduced sperm motility and induced cytotoxicity (Pant et al., 2011). *In vitro* grown human testes explants showed that DHEP inhibited testosterone synthesis (Desdoits-Lethimonier et al., 2012).

Human studies have confirmed the *in vitro* findings, linking phthalate exposure to reduced sperm motility in males from infertile couples (Duty et al., 2003). There was a weak correlation between DNA damage, sperm motility, and morphology and phthalate exposure (Hauser et al., 2007; Liu et al., 2012), but an inverse relationship was found between MEHP metabolite increase and testosterone and estradiol levels (Hauser et al., 2007). Beyond phthalates, BPA exposure impacts testosterone levels in men and women’s plasma, whereas upstream hormones like FSH are downregulated, probably due to the endocrine disrupting activity. Mendiola and colleagues (2010) found that BPA levels were adversely linked with androgen concentrations, such as testosterone, in 360 fertile men. However, Meeker and co-workers (2010) reported no significant trends between urinary BPA levels and oestradiol/testosterone index, lower sperm counts, or DNA damage, contrary to Mendiola et al. (2010) findings.

Men exposed to BPA at work had worse sperm quality indicators (Li et al., 2011). Notably, the median urine concentration was nearly 70 times lower than the USEPA’s recommended daily intake, whereas the control group had exposure dosages approximately 2,000 times lower. This research showed that BPA can be detrimental at amounts well below the USEPA’s recommended daily dosage (Li et al., 2011). BPA was also linked to decreased sperm count, sperm morphology, sperm motility, and DNA damage. BPA was linked to increased FSH, decreased inhibin B, and a poorer oestradiol: testosterone ratio (Meeker et al., 2010). In a study of 375 fertile males, BPA was linked to lower free androgen index and increased testosterone (Mendiola et al., 2010). Similar results for PCBs, a structurally related class of contaminants, have been reported. Petersen and co-workers (2015) tested 266 fertile men for PCB exposure, hormone levels, and sperm quality. The ratio of testosterone/estradiol, FSH, and SHBG were strongly linked with serum PCB levels, but not with semen parameters including volume, sperm concentration, total sperm count, morphology, or abstinence period.

#### 3.2.2 EDCs and testicular pathology

Studies in Denmark and Norway revealed that roughly 1% of males will get testicular cancer during their lifetime (Skakkebaek et al., 2007). Between 1998 and 2002, the incidence of testicular cancer in men in Norway was approximately 5 times greater than in Spain (Gonzalez et al., 2008; Chia et al., 2010). This study points to a higher frequency in developed countries than in developing ones. Initially, these variations were attributed to the populations’ ethnic diversity and immigration from nations with high cancer morbidity rates (Nordkap et al., 2012). From 1997 to 2001, the prevalence of cryptorchidism increased significantly in Denmark (Boisen et al., 2004), while it decreased in England and Finland (Boisen et al., 2004; 2005). These geographic trends were followed by specialized research that linked maternal–lifestyle factors like higher painkiller usage, particularly paracetamol and ibuprofen, to cryptorchidism in offspring (Thorup et al., 2006; Jensen et al., 2007). Environmental pollutants like phthalates have been linked to poor semen quality, testicular cancer, cryptorchidism, and hypospadias (Nordkap et al., 2012). The “so-called” testicular dysgenesis syndrome theory is based on clinical analysis of these three male reproductive system disorders. Testicular cancer and poor semen quality have been postulated as explanations for the abnormal gonad differentiation and subsequent development of Leydig cells that release testosterone and initiate masculinization of the male foetus (Sharpe et al., 2003; Sharpe, 2006). To date, no definitive link between pesticide as a form of EDC exposure and testicular cancer has been established, while a genetic background and environmental factors have been linked to lower sperm counts and the disease. Moreover, the impacts may not be identified for two to three decades after foetal exposure. This emphasizes the value of human population epidemiological evaluation studies and limitations of clinical research (Publicover et al., 2007; Nordkap et al., 2012).

## 4 Mechanism of action of EDCs

The mode of action of EDCs on human reproduction is complex and can vary depending on the type of EDCs and the exposure level. Some EDCs have a chemical structure similar to natural hormones in the body. These can mimic the actions of hormones, such as estrogen and androgen, and bind to their receptors, causing the same response as the natural hormone. This can disrupt the normal hormone balance and lead to reproductive disorders. EDCs can also inhibit the production or action of hormones in the body, leading to reproductive dysfunction. For example, some EDCs can inhibit the production of testosterone or inhibit the conversion of testosterone to estrogen, leading to decreased fertility in men. They may alter gene expression in reproductive tissues, leading to changes in cellular processes and hormone regulation. EDCs can also induce epigenetic changes, which can alter gene expression and result in reproductive disorders. For example, exposure to certain EDs during critical periods of development can cause DNA methylation changes, which can lead to infertility and other reproductive disorders. Moreover, EDCs are potent inducers of OS, which can damage cells in the reproductive system and lead to infertility (Ghosh et al., 2022; Pallotti et al., 2022). The following sub-sections discuss the most common modes of action of EDCs in affecting human reproductive health.

### 4.1 Modulation of hormone receptors and enzyme actions

EDCs are detrimental because they disturb the typical mechanisms that maintain a balance of hormones, which facilitate tissue growth and advancement. When it comes to the reproductive system, EDCs hinder the ability of hormones to bind to their intended receptor, particularly the androgen receptor (AR) or the estrogen receptor (ER) (Monneret, 2017). EDCs have the ability to produce two different reactions upon attaching to a receptor: a hormone-based response, referred to as an agonistic effect, or the absence of a hormone-based response, known as an antagonistic effect. Studies indicate that the estrogen receptor subtypes, ERα and ERβ, exhibit agonistic effects when exposed to the organochlorine pesticide MTX, which was created as a replacement for DDT. In contrast, the androgen receptor subtypes demonstrate antagonistic effects when exposed to MTX (Gaido et al., 2000; Waters et al., 2001; Mrema et al., 2013). Tetrachlorodibenzo-p-dioxin (TCDD) is a hazardous environmental pollutant that has been shown to act as an inhibitor or antagonist of hormone synthesis. This substance also exhibits anti-androgenic effects. Another example of a chemical compound with endocrine-disrupting properties is the pesticide endosulfan, which possesses estrogenic activity. Endosulfan has been demonstrated to induce ovarian regression both *in vitro* and *in vivo* (Senthilkumaran, 2015). Endosulphan competes with estradiol (E2) for estrogen receptor binding but has a lower affinity (Lemaire et al., 2006). As a result, sex-specific gene expression is affected. On the other hand, the expression of various male reproductive genes, such as testis-related transcription factors (Sox9a and wt1), was suppressed by endosulfan (Rajakumar et al., 2012), while BPA is non–steroidal with binding affinity for estrogen receptors, ERα and Erβ (Welshons et al., 2003).

EDCs can impact the activity of enzymes involved in the regulation of steroidogenesis and hormone metabolism, as well as hormone-receptors (Figure 1). Examples include phthalates, a kind of pesticide that has anti–androgenic characteristics by lowering testosterone production in Leydig cells as a result of direct CYP17 suppression (Foster, 2005). EDCs may also inhibit 5-α reductase, an important enzyme for synthesis of dihydrotestosterone from testosterone, thereby facilitating masculinization of gentalia (Fisher, 2004a). Thiophosphates, a type of organophosphorous insecticide, inhibit the P450 enzymes CYP3A4 and CYP1A2 in the liver, which are involved in the metabolism of estrone and testosterone (Usmani et al., 2003; 2006).

EDCs have also been shown to have an effect on the expression of hormone receptors in experimental animals. In Sprague–Dawley rats, it was shown that prenatal exposure to DEHP caused enhanced mineralocorticoid receptor mRNA and protein expression in adult interstitial Leydig cells, while toxicological studies in rats revealed that BPA produced dysregulation of steroid receptors and co–regulators (Martinez-Arguelles et al., 2009; Skinner et al., 2011).

It is worth noting that EDCs have multiple hormone–binding properties, regardless of whether or not they bind to hormonal receptors. For instance, DDT is an estrogen receptor agonist, but one of DDT’s metabolites is an anti–androgen (Kelce et al., 1995). In addition to its estrogenic and androgenic properties, BPA is a thyroid hormone antagonist (Moriyama et al., 2002; Wetherill et al., 2005).

Several investigations have shown that EDCs alter deiodinase activity, decrease thyroid hormone metabolism, restrict iodine absorption by thyroid cells, competitively inhibit thyroid transport protein TTR, and antagonize complexes arising from the thyroid hormone responsive elements (TREs) (Butt and Stapleton, 2013; Noyes et al., 2013). The homological resemblance between thyroxine (TH) and particular TH-disrupting chemicals (TH-EDCs), such as PCB metabolites, dioxin (PCDD), and brominated flame retardants, results in binding to the high-affinity TH transport protein, transthyretin TTR, thereby obstructing T4-TTR binding (Grimm et al., 2013). Since TH governs lipid and glucose metabolism, the consequences of EDCs surpass perturbation of typical TH levels to influencing the lipid profile and glucose homeostasis of those exposed. For instance, in rats, BPA was discovered to promote the formation of visceral adipose tissue and cause hyperlipidemia (Miyawaki et al., 2007). Lastly, certain TH-EDCs disrupt and/or interplay with the nuclear receptors of hormones that operate through nuclear receptors, such as sex hormones.

EDCs possess the ability to induce alterations in the expression of enzymes involved in steroid and sex hormone metabolism at the molecular level through the initiation of corresponding transcription via binding to nuclear receptors. Specifically, organochlorine pesticides and dioxins are known to interact with the aryl hydrocarbon receptor (AhR) and promote the transcription of CYP1 genes, which subsequently catalyze the hydroxylation of E2 (Park et al., 2017). Previous studies have demonstrated that TCDD can also interfere with ovarian steroidogenesis, resulting in reduced levels of progesterone, androstenedione, testosterone, and E2 *in vitro* and *in vivo* (Park et al., 2017). Moreover, MTX, an organochlorine pesticide, can obstruct ovarian steroidogenesis and suppress the production of E2, testosterone, and androstenedione by downregulating the expression of enzymes including CYP19α1, CYP17α1, CYP11α1, and 17-β-hydroxysteroid dehydrogenase (Basavarajappa et al., 2011). The MTX metabolite 2,2–bis(p-hydroxyphenyl)–1,1, 1–trichloroethane (HPTE), which reduces FSH-stimulated progesterone and estrogen synthesis by lowering CYP11α1 and CYP19α1 expression, has a comparable anti–estrogenic action (Zachow and Uzumcu, 2006).

EDCs have been demonstrated to stimulate the expression of endogenous or xenobiotic-metabolizing enzymes, leading to alterations in their metabolic pathways within the human body. One example of this is the phthalate class of EDCs, which has been observed to induce the AhR and the enzyme CYP1B1 responsible for metabolizing E2 (Chen L et al., 2013). Phthalates, like MTX and TCDD, reduce E2 and DEHP levels in the body by downregulating CYP19α1 expression (Gupta et al., 2010; Hannon et al., 2015).

### 4.2 Alterations at the epigenetic level

Epigenetic impacts of EDCs are heritable changes in gene function that occur in the absence of DNA sequence modifications. Notably, transcription factors that repress or increase the transcription of certain genes mediate epigenetic changes. DNA methylation, post–translational changes of histone proteins (histone acetylation and deacetylation), and non–coding RNA are the main epigenetic processes (Jirtle and Skinner, 2007). Because DNA methylation influences the binding of transcription factors to DNA, it usually results in a reduction in gene expression. The structure and function of chromatin may be altered by post-translational changes of histone proteins at certain amino acid residues, such as lysine (Turner, 2009).

It is generally believed that acetylation of histones causes transcription to be activated as a result of chromatin relaxation, whereas deacetylation causes gene silencing and transcriptional repression. Non–coding RNAs are transcripts of sequences that do not code for proteins yet regulate gene expression in both the cis and trans directions (Chang et al., 2006). They play a role in X–chromosome inactivation, genomic imprinting, and embryonic patterning and differentiation, among others.

Studies in experimental animals have shown that EDCs like diethylstilbesterol (DES) can stimulate the expression of early genes in neonates, such as c–fos, c–jun, c–myc, and lactoferrin, which are all elevated in childhood (Nelson et al., 1994). This was accompanied by hypomethylation of the lactoferrin gene promoter region in the adult uterus (Li et al., 1997), however no such pattern of methylation was detected when the animals were subjected to the same period during adulthood. Hypomethylation of exon–4 of the fos gene, which was identified in later research, was assigned to the exact cause (Li et al., 2003). DES is also linked to hypermethylation of the Hoxa–10 gene’s promoter and intron regions, which leads to anterior reproductive tract alterations (Block et al., 2000). Importantly, EDC interference with epigenetic control of numerous genes has been linked to additional upstream cell signaling networks, such as the ER and PI3K/Akt pathways (Falck and Forsberg, 1996; Bredfeldt et al., 2010).

The organochlorine pesticide methoxychlor has been linked to epigenetic alterations in the ovary (MTX). In a 2009 study, Zama and Uzumcu (2009) employed bisulphite–sequencing PCR and methylation–specific PCR to show that MTX produced hypermethylation in several CpGs in the ER promoter sequences. The degree of DNA methylation in promoter regions appears to be age–dependent, as the aforementioned genes showed a distinct methylation pattern in neonatal ovaries exposed to EDCs (MTX, DES) (Tang et al., 2008; Zama and Uzumcu, 2009). In terms of the gene targets methylated by MTX, genome–wide methylation investigations have revealed that transcription factors and ribosomal proteins make up majority of potential genomic areas. PTEN and IGF are two important signaling pathways that have been found to be hypermethylated by MTX (Zama and Uzumcu, 2009).

### 4.3 Alternative mechanisms of action

There have been several different postulated mechanisms of action for EDCs in terms of disrupting normal reproductive system development that are not directly connected to the control of hormone functions but can indirectly also affect the hormonal milieu. Exposure to EDCs has been shown to affect Sertoli cells in two ways: by inducing apoptosis in spermatocytes and by increasing expression of apoptotic proteins (Zhang et al., 2019). Sertoli cells play a vital role in supporting the development of spermatocytes, including the absorption of excess cytoplasm and acceleration of testosterone-linked spermatogenesis. Failure of Leydig cells to produce testosterone results in the collapse of the testosterone-bound androgen receptor-mediated gene transcription, that is crucial for spermatogenesis. Studies have shown that EDCs, particularly BPA, can inhibit ATP production, likely due to mitochondrial disruption, leading to impaired sperm motility (Rahman et al., 2017). EDC exposure creates an abnormal hormonal milieu that can result in aneuploidy in sperm and potentially induce transgenerational effects. For example, the chemical MEHP was shown to negatively impact ovarian follicle by increasing the production of reactive oxygen species (ROS) and the level of oxidative stress, while simultaneously downregulating the expression of the enzymes glutathione peroxidase (GPx) and superoxide dismutase 1 (SOD1) (Wang et al., 2012). The induction of free radicals as a result of lead exposure has the potential to exert negative effects on the survival, number and motility of sperm cells, in addition to elevating DNA fragmentation and chemotactic responsiveness towards spermatozoa-ovum fusion. These factors could collectively impede the fertilization process (Gandhi et al., 2017). DBP suppresses the expression of cyclins E1, A2 and B1, resulting in cell cycle arrest and consequent suppression of antral follicle development (Craig et al., 2013). In addition, BPA was shown to induce apoptosis, via a caspase 3-mediated mechanism in female rat follicles and to regress the luteum development, that is considered as the main cause of infertility (Lee et al., 2013).

### 4.4 Metabolism of EDCs

Metabolism of EDCs might be a primary contributor to the adverse health impacts. Organochlorine chemicals, such as DDT and dioxin, are generally difficult to metabolize due to the large amount of unconjugated chlorine atoms in their structure. As a result, such chemicals have a lengthy half-life in the human body, which contributes to the ongoing stimulation of negative effects (Androutsopoulos et al., 2013). Organochlorine chemicals, such as pollutants and pesticides, can activate phase I and phase II metabolizing enzymes such as UGT1A6, NQQ1, CYP1A1, and CYP1B1 via activating the AhR (Androutsopoulos et al., 2009). As a result, conversion of polycyclic aromatic hydrocarbons, nitrosamines, and other environmental pollutants to physiologically active metabolites is facilitated (Androutsopoulos et al., 2013; Aoyama and Chapin, 2014). In the case of the EDC methoxychlor, the active metabolite hydroxyphenyltrichloroethane has been demonstrated to have roughly 100-fold higher estrogenic activity and efficacy in animals exposed to methoxychlor than the parent molecule (Aoyama and Chapin, 2014). Methoxychlor is mostly demethylated and hydroxylated by cytochrome P450 enzymes such as CYP3A4, CYP3A5, and CYP2B6. In contrast to MTX, BPA has been found to be converted to the metabolites bisphenol A-4-sulfate and bisphenol A-4D-glucuronide via sulfation and glucuronidation processes, respectively, which do not have activity on endocrine receptors (Skledar and Masic, 2016). Phase I enzymes have also been used to document hydroxylation reactions for BPA. The 5-hydroxybisphenol A metabolite is thought to be less efficient than the parent molecule (Elsby et al., 2001). Phthalates are metabolized in a similar way to BPA, where detoxification of the compound is mediated via glucuronidation reactions, whereas paraoxonase 1 (PON1) mediates the metabolism of organophosphorus pesticides to the common metabolite dialkyl phosphate (DAP). The cytochrome P450 enzymes CYP2C19, CYP2B6, and CYP3A4 perform an initial desulfuration process to produce the appropriate oxon metabolite, which is then hydrolyzed via PON1 to produce DAP (Androutsopoulos et al., 2013). Bulk of human research emphasized primarily on detecting OPs metabolites over evaluating their biological activities, OPs metabolites maintain a portion of the parent compound’s action.

## 5 Toxicokinetics of cocktail mixtures

### 5.1 Mechanism(s) of interaction of EDCs

Humans are subjected to exposure to environmental chemicals that are present in infinite number of combinations. This makes it difficult to exactly assess the actual impact or risk of an individual chemical on male reproductive health in the laboratory setup. Here comes the importance of studies that consider assessing the impact of various possible combinations of EDCs on male reproductive health (World Health Organization, 2009). Recent research has identified four types of combination effects or interactions.

#### 5.1.1 Dose addition

In such combinations, toxicity is generated by the same mechanism of action. Even though individual chemical exposures are too minimal to evoke a response, if one is exposed to a combination including a large cocktail of compounds with the same mechanism of action, a response may be generated. As per the dose addition, substances with the same mechanism of action are considered following the mentioned principle (World Health Organization, 2009).

#### 5.1.2 Response addition

Individual substance exposure must be sufficient to elicit a reaction without the participation of the other substance in this interaction since both chemicals have separate mechanisms of action. By substance, a neurotoxin administered in combination with another hepatotoxin induces neurotoxicity as well as liver toxicities, with the same outcomes as if each toxin was administered individually. When a large number of substances are exposed, such interactions become insignificant (Kortenkamp, 2007; World Health Organization, 2009).

#### 5.1.3 Synergism

When at least one of the components has a positive interaction with the biological system, this is known as a positive interaction. It happens when the combined effect is larger than the sum of the individual activities of each component at the same level of exposure as that experienced by the mixture as a whole. When one drug modifies the metabolism of another potentially more dangerous substance in order to enhance its active form of the toxic internal dosage of the substance or systemic exposure this is called a toxicokinetic interaction. Such interactions have the potential to increase the activity of the hazardous chemical in the body (Kortenkamp, 2007).

#### 5.1.4 Antagonism

Both substances are necessary and must be present at active concentrations in order for antagonism to be effective. It is conceivable for toxicokinetic and toxicodynamic interactions to be antagonistic to one another. These interactions may have the effect of decreasing the toxicity of the active principle(s). As the name implies, antagonism is the competition between two compounds for efficacy, with one chemical having a low efficacy and the other having a high efficacy, which are known as partial and full agonist, respectively (Kortenkamp, 2007; World Health Organization, 2009).

To evaluate the likelihood of combination toxicity in connection to the risk assessment of environmental contaminants and their negative effects on health, a large quantity of information is required. Determining the interaction between chemicals is highly challenging due to the limited approaches available (Chen M et al., 2013). No evidence of a mechanism of interaction between heavy metals and pesticides has been found. Pesticide and heavy metal toxicity may interact, amplifying or lowering the harmful impact of the subsequent xenobiotic, and perhaps accounting for the total effect of both xenobiotics. At individual concentrations, heavy metal and pesticide toxicity processes have been widely studied. The mechanism of synergistic toxicity of xenobiotics, on the other hand, has not been adequately examined. Research that reveals how heavy metals and pesticides interact and impact each other’s toxicity has yet to be completed. As a result, more study in this area is required.

Pesticides and heavy metal exposure provide a significant health risk to individuals under biological and environmental settings (Chen L et al., 2013; He et al., 2015). For genotoxic and carcinogenic substances, non-linear dose-response correlations may exist. To make the dose-response evaluation, the available dose-response data should be employed. Many scientists have studied the biotransformation of heavy metals, pesticides, and other EDCs separately. However, in terms of a combined study as well as the methods by which they impact each other’s toxicity, there has not been adequate research.

### 5.2 Concentration-based strategy

Expertise in selecting exposure concentration is a critical parameter that contribute to the predicted combined effects in mixture toxicity studies (Cleuvers, 2004; Dalla Bona et al., 2014). Various techniques have been developed and used for different chemical exposure groups employing different concentration gradients (Lin et al., 2005; Dietrich et al., 2010; Dou et al., 2011; Ginebreda et al., 2014). Combined outcome may be influenced by the concentration ratio of the mixture components after the concentration levels have been determined. The Climax Hypothesis, which was formed from a broader concept of “isobolograms,” argued that the ratios of separate chemicals might affect the cumulative effects of chemical combinations (2010; Dou et al., 2011; Lin et al., 2005). According to prior research, combined effects can be detected at an equitoxic ratio of 1:1, though the combined effects are dependent on the toxicology and bioavailability of the chemical combination (Escher and Fenner, 2011; Ginebreda et al., 2014). Chen et al. (2014) additionally treated the six priority PAEs binary combinations to marine medaka (*Oryzias melastigma*) and zebrafish at an equivalent toxic ratio (1:1) to investigate related estrogenic effects. Likewise, male rats were given quinary mixes (1:1) of six priority PAEs and glycerin monostearate (GMS) for 15 weeks to study the behaviour of steroidogenic proteins (Gao et al., 2018). Furthermore, rather than utilizing high dosages that do not occur in the natural environment, the exposure evaluation should be based on the environment-relevant-concentrations (ERCs) of the chemicals (Kabir et al., 2015).

Exposure concentration for chemical mixtures has two main concepts: effective and nominal concentrations (Hadrup et al., 2013). The effective concentration (ECx) is the concentration at which the highest effects may be detected (Kabir et al., 2015). The exposure effect (EC) might be 10%, 50%, or 90% depending on the chemical kinetics, transformation, absorption effects, and catabolism (Escher and Fenner, 2011; Ginebreda et al., 2014). EC50 is the graded or optimal dose-response curve parameter that reflects the concentration of a chemical necessary to have 50% maximal effect. It is determined using statistical methods such as hillslope, logit, and probit analyses (Ge et al., 2014; Kienzler et al., 2016; Miller et al., 2017). The hillslope technique is now the most often used to determine EC50 (Hadrup et al., 2013; Pradhan et al., 2018). Hillslope is equal to 1 when the receptor chemical binds to one site independently, while it is higher than 1 when the receptor or ligand has many binding sites with positive cooperativity (Hadrup et al., 2013). Negative cooperativity occurs when many binding sites with varying ligand binding affinities are accessible (Hadrup et al., 2013). Also, the concentration impact is a major aspect in assessing danger in chemical combinations (Ge et al., 2014). These models rely on the dose-receptor (EC50) connection, which is also used to estimate combination toxicity (Dalla Bona et al., 2014; Ge et al., 2014). The steroid production in H295R cells was studied using quaternary antibiotic combinations with EC50 values (Hadrup et al., 2013). It also depended on the EC50 value for PAEs and metal co-exposure (DBP, DEHP, Cu) (Huang et al., 2016). Furthermore, a similar concentration gradient was employed to investigate the disruption in the detoxification-metabolic pathways in zebrafish embryos (*in vivo*) after exposing binary sulfonamides (SAs) combinations to *in vitro Acinetobacter* Tox2 to estimate the EC50 value (Hamid et al., 2020). It is important to consider the amount of chemical kinetics (absorption, distribution, metabolism, and excretion) and toxicodynamics (interaction with receptor sites/target organs) that reaches the site of action following exposure (Pal et al., 2010).

### 5.3 Determination of combined effect of EDCs by mathematical models

Diverse mathematical modeling approaches are used to identify the combined impacts of environmental samples and laboratory compounds. Modeling techniques rely on input parameters, exposure models, and knowledge of chemical mixture volume and pattern. CA, IA, and CI are mathematical models used to evaluate chemical combinations both retrospectively and prospectively. In 1926, Loewe proposed the concentration addition/additive dosage model, which assumes that all compounds in a combination possess same MoA and biological target site, regardless of their individual efficacy (Cedergreen et al., 2008). The CA model is determined via the equation: (cA/ECxA) + (cB/ECxB), where cA = chemical A concentration, ECxA = effective concentration of A that leaves 50% of the population, cB = chemical B concentration, ECxB = effective concentration of chemical B that kills 50% of the population (Cedergreen, 2014; Huang et al., 2015). However, in 1939, Bliss made the assumption that chemical mixes have a different MoA or that compounds in a combination tend to react on various target sites (Hadrup et al., 2013; Chen et al., 2015; Huang et al., 2016). It is phrased as follows: C_AB_ = C_A_ + C_B_ + C_AB_, where C is the mixture concentration of A, B, and AB (Hadrup et al., 2013). Both models are classical models that help quantify the chemistry of mixtures (Jensen and Sverdrup, 2002; Hadrup et al., 2013). Chou (1976) created the CI iso-bologram equation in 1976, which follows the median effect concept established from mass action law. The CI model unambiguously links dose-effect mechanism of action with independent inhibition (De Liguoro et al., 2010). The process is mechanized using CompuSyn software, which automates the modelling of synergism and antagonism between compounds at various concentrations and effect levels (Chen et al., 2015; Hamid et al., 2020). Moreover, compared to other models, the CI model has the highest fit or predictive capacity (Gonzalez-Pleiter et al., 2013; Chen et al., 2015).

On comparing the experimental and modelled values of synergistic, additive, and antagonistic toxicity in most of the combined toxicity studies, toxicants acting on the same target at separate locations, overlapping sites, or targets in the same pathway have been found to show synergistic effects (Jia et al., 2009; Ginebreda et al., 2014). Opposite or repulsive effects are described as antagonistic effects (Backhaus et al., 2003; Chen et al., 2015). Additivity shows how chemicals “act together” to generate effects without amplifying or reducing one other’s impact (Kortenkamp, 2007; Cedergreen et al., 2008). More than single/individual pollutants, these impacts might have a negative influence on aquatic ecosystems (Denton et al., 2003).

A comparable chemical MoA was shown to be more trustworthy in previous ecotoxicological research (Cedergreen, 2014; Fang et al., 2016). Tests on amphetamine and quinolone combinations revealed that they are hazardous to each other (Backhaus et al., 2000; Jensen and Sverdrup, 2002; De Liguoro et al., 2009; Dias da Silva et al., 2013). An antibiotic’s and pesticide’s binary dose-response surfaces with distinct molecular targets were compared by Cedergreen et al. (2008) to assess model correctness. However, neither model predicted more than 20% of the mixes effectively (Huang et al., 2011; Zou et al., 2013). To cope with mixes with high exposure levels, is important to choose the model that best extrapolates trends and most closely matches actual environmental contamination scenarios.

In H295R cells, Hadrup et al. (2013) reported a substantial drop in testosterone levels when PPCPs and phthalates were combined. A second effect of testosterone on the neurological system is its local aromatization into E2 (Madureira et al., 2012). NSAIDS and sulfonamides in binary and quinary combinations also had additive effects (De Liguoro et al., 2009; Fang et al., 2016; Bialk-Bielinska et al., 2017). For the male reproductive system, the IA model outperformed the integrated addition model in terms of synergy (Hao et al., 2013). There are synergistic or additive effects with binary BPA + DBP mixes that upregulate AR, gonadotrophin-releasing hormone receptor (GnRHR), and progesterone hormone receptor (PRs) expression levels in male rats (Zhang et al., 2013). Following synergistic effects, additive or antagonistic effects were seen in most mixes from known references (IA, CA, CI). In other words, increased synergism may enhance the EDC combinations (Katsnelson et al., 2015; Hamid et al., 2020).

## 6 Mixtures of EDCs: direct and indirect effects on fertility

As previously discussed, the impact of EDCs on overall health and reproduction is multifaceted (Ghosh et al., 2022). EDCs, such as bisphenol A, phthalates, and persistent organic pollutants, can interact with endocrine receptors, leading to hormonal imbalances, altered homeostasis, and developmental abnormalities. These disruptions may result in reproductive disorders including reduced fertility, abnormal gamete development, and compromised offspring viability (Rider et al., 2010; Ghosh et al., 2022). Additionally, EEDs have been linked to metabolic dysregulation, increased susceptibility to endocrine-related cancers, and neurodevelopmental deficits. The cumulative effects of EEDs pose considerable risks to human health, necessitating further research and intervention strategies to mitigate their consequences (Luijten et al., 2023).

## 6.1 Mixtures of estrogenic chemicals

Estrogenic compounds have been the focus of the bulk of EDC research therefore it is no surprise that they have been the subject of majority of EDCs combination studies. Although early research focused mostly on binary mixtures, work done after 1998 has made important advances to the study of multicomponent combinations including 3, typically 5, and up to 12 estrogenic compounds (Kortenkamp and Altenburger, 1998). “Estrogenicity” is a term that may be defined in a variety of ways (Kortenkamp, 2007). The phrase refers to a chemical’s potential to elicit reactions comparable to those of 17-β-estradiol (E2), such as cornification of the vaginal epithelium and uterine cell proliferation, on a functional and physiologic level (Kortenkamp, 2007). The involvement of estrogens in breast and ovarian cancer is of toxicologic importance, because E2 and synthetic estrogens are known human carcinogens. Further classifications that relate to particular stages at various molecular levels have resulted from advances in our knowledge of estrogens’ method of action (Payne et al., 2000; Kortenkamp, 2007). These criteria help to organize the information on estrogen combinations, e.g., “estrogenicity” might refer to agonist or antagonist affinity for the ER, capacity to trigger estrogen-dependent gene expression, or promotion of cell proliferation in ER competent cells (Payne et al., 2000; Rajapakse et al., 2002).

### 6.2 Antiandrogenic EDCs

Androgens control male sexual differentiation and early postnatal development. During this time span, chemicals that inhibit androgen activity might cause reproductive abnormalities. Physiologic alterations for reproductive development, such as retained nipples, modifications in anogenital distance and weight changes of sexual organs and associated glands are often researched end goals. These effects can be caused by androgen antagonists at steroid receptors or by suppressing testosterone production in Leydig cells (Nellemann et al., 2003; Fisher, 2004b). Antiandrogens are defined as AR antagonists, while a broader definition has been offered that includes preventing the synthesis and activation of testosterone and its precursors (Gray et al., 2001). Nellemann et al. (2003) reported that AR antagonists, vinclozolin, and procymidone block testosterone to AR binding, via isobole technique. In castrated, testosterone-treated male rats, a 1:1 combination of fungicides to castrated altered reproductive organ weights, androgen levels, and AR-dependent gene expression. Birkhoj et al. (2004) used the isobole approach for three-component pesticide combinations comprising deltamethrin, methiocarb, and prochloraz. *In vitro*, an equimolar combination of the three insecticides inhibited AR activation. The investigators found weight changes in the adrenal gland and levator ani, as well as changes in gene expression of AR-associated genes, when these three compounds were given to castrated testosterone-treated rats. However, the cumulative effects of all five substances could not be determined.

In the Hershberger assay, procymidone and vinclozolin (AR antogonists) combined to reduce ventral prostate and levator ani weights (Gray et al., 2001). Giving pregnant rats procymidone with dibutyl phthalate, an androgen synthesis-inhibitor, increased the incidence of hypospadias in male offspring during gestation days 14–18 antagonizing the effects of vinclozolin and testosterone propionate on male sexual differentiation. Androgen-dependent tissues were altered in dose-additive way when butylbenzylphthalate (BBP) and linuron (LIN) were combined (Hotchkiss et al., 2004). When male offspring of female rats were given di-(2-ethylhexyl) phthalate plus di-(2-ethylhexyl) adipate, the effects were similar to those of each chemical alone.

### 6.3 Thyroid-disrupting EDCs

Thyroid-disrupting compounds are the least investigated EDCs when compared to estrogens and antiandrogens (Kortenkamp, 2007). By interfering with related regulatory enzymes, thyroid-disrupting drugs can change the functions, morphology, and secretions of thyroid glands (Wade et al., 2002; Desaulniers et al., 2003). Thyroid hormone levels in the bloodstream often change as a result. Thyroid hormone levels can be influenced by a wide range of compounds in different ways. PCBs, PCDDs, and PCDFs are considered to reduce the levels of thyroid hormones by increasing the activities of liver enzymes which in turn mediate glucuronidation of thyroxin (T4). Majority of the investigations on thyroid-impairing impacts have looked at the effects of EDCs mixes rather than individual mixture components, which makes assessing combination effects in terms of synergism, additivity, or antagonism more difficult. Wade et al. (2002) studied the effects of a mixture of organochlorines and two heavy metals on thyroid histology in rats. Desaulniers et al. (2003) had later used the 2,3,7,8- tetrachloro-p-dibenzodioxin (TCDD) equivalents approach to demonstrate the impacts of 16 PCDDs, PCBs, and PCDFs on circulating levels of T4. Crofton et al. (2005) conducted a detailed investigation of a combination of 18 polyhalogenated hydrocarbons to see if their combined impact on lowering T4 levels is dose-additive. Individual mixture components were given to young female rats for 4 days, and dose–response relationships with changed T4 levels as the end points were recorded. The dose-additive response to a combination of all 18 compounds was predicted using this data. The chemical levels reported in fish, breast milk, and other diet sources were used to determine the mixing ratio. The dose-additivity model predicted effect dosages that were two to three times greater than actual responses. This difference was statistically significant, indicating that the combined impact of all polyhalogenated EDCs in this model work synergistically (Kortenkamp, 2007).

## 7 Role of antioxidants on combined EDCs induced reproductive toxicity

### 7.1 Role of endogenous antioxidant system in reducing toxicity

The endogenous antioxidant system contains both enzymatic and non-enzymatic antioxidants. Some antioxidant enzymes known to be involved in cellular redox processes include glutathione peroxidase (GPx), glutathione-S-transferases (GST), superoxide dismutase (SOD), peroxidase, catalase, and GSH. Endogenous antioxidants (SOD, GPx, catalase) are critical in the fight against oxidative stress caused by heavy metals, pesticides, and combinations of the two. EDCs-induced toxicity is caused by an increase in the production of free radical molecules such as the superoxide radical (O_2_^•–^), hydroxyl radical (•OH), nitric oxide (•NO), and peroxynitrite (ONOO^−^). SOD and catalase reduce oxidative stress caused by xenobiotics, especially heavy metals, pesticides, other EDCs and their mixtures, by diminishing free radical levels by the converting the highly reactive ROS and RNS (reactive nitrogen species) to inert molecules (Meli et al., 2020). The metalloenzyme, SOD, catalyzes superoxide radical dismutation into hydrogen peroxide, as a defense mechanism against oxygen toxicity (Natvig et al., 1996). In this mechanism, GPx is also involved, inactivating hydrogen and lipid peroxides to eliminate free radical species [208]. By preventing phospholipid peroxidation, GPx shields membranes from oxidative damage. It is also capable of metabolizing hydrogen peroxide (Ighodaro and Akinloye, 2018). GST catalyzes GSH in conjugation with toxic compounds, to form less toxic product with higher water-solubility. Moreover, as the end products are less biologically active, they easily get excreted and thereby also neutralize free radicals that may cause membrane damage (Raijmakers et al., 2001). In several species, catalase (an enzyme involved in hydrogen peroxide metabolism), is functionally replaced by peroxiredoxin (Prx) and GPx (Flohe et al., 2003).

### 7.2 Role of antioxidant supplements in reducing reproductive toxicity

Antioxidants are chemicals that suppress over-generation of intracellular ROS thereby mitigating oxidative stress and oxidative damage to the tissues. Several studies have shown that antioxidants such as vitamin C, vitamin E, N-acetylcysteine, lipoic acid, gallic acid, and ginger extract, can reduce or prevent EDC-induced damage (Korkmaz et al., 2010; El-Beshbishy et al., 2011; Jain et al., 2011; Amraoui et al., 2018; Mohammed et al., 2020). Antioxidant supplementation protects spermatozoa quality, motility, vitality, and morphology (Mortazavi et al., 2014). Antioxidants minimize oxidative stress in spermatozoa during cryopreservation and maintain spermatozoa motility and fertility (Bisht et al., 2017). Various antioxidants, alone or in combination, have been demonstrated to be effective in reducing EDCs toxicity in male and female reproductive systems.

Vitamin C, vitamin E and glutathione, have been shown to improve sperm functions (Fanaei et al., 2014; Shah et al., 2017; Srivastava and Gupta, 2018). These micronutrients prevent impairment of spermatozoa motility and disruption in acrosome response that are triggered in EDCs-exposed spermatozoa. Vitamins also enhance the intracellular ATP production. When ROS and their metabolites are produced in excess, they may lead to permanent cell damage by affecting spermatozoa enzymatic systems, DNA, lipids, and proteins (Agarwal et al., 2014). EDCs, such as BPA have been shown to impair fertilization and early embryo development, whereas vitamin E (2 mM) and glutathione (5 mM) could counteract adverse actions of EDCs (Rahman and Pang, 2019). One of the most important endogenous antioxidants, glutathione, is recycled by vitamin C and E (Dwivedi et al., 2020). It functions as an electron donor in a variety of activities and is recycled intracellularly at the cost of NADH or glutathione. Vitamin E is a category of lipophilic vitamins that have antioxidant and cytoprotective properties (Galli et al., 2017).

Natural compounds such as herbals and phytochemicals should be explored among other preventative or therapeutic treatments. Gallic acid, a polyhydroxyphenolic molecule, is mostly found in free form or as a component of hydrolyzable tannins in plants, vegetables, and certain fruits. This compound shows antioxidant actions by metal ion sequestration and ROS scavenging (Badhani et al., 2015). Gallic acid (20 mg/kg in maize oil) was recently reported to have positive impact upon testicular oxidative stress in adult rats, induced by chronic exposure to EDCs, like BPA (Olukole et al., 2020). A variety of biological activities are demonstrated by the fungus *Cordyceps militaris*, which is used in traditional Chinese medicine to treat infertility. Cordycepin and polysaccharides content, attributes for the antioxidant activities of *C. militaris* (Yue et al., 2013).

## 8 Conclusion and future perspective

Combined toxicity of EDCs on human reproduction is a complex phenomenon, which needs thorough understanding to facilitate incorporation of these concepts into regulatory frameworks. There are several conceptual gaps and uncertainties to consider regarding the combined effects of EDCs on reproductive functions. One of the primary challenges is selecting the experimental design, that is, the criteria to be used to pick the mixture components. A few factors including cost, time, and accuracy should be considered to provide a realistic representation of environment-relevant concentration of the EDCs mixtures. Aside from studies on the parent compound mixtures, investigations on transformation products, such as metabolites produced by photo-degradation and biodegradation, are rare. Furthermore, because the transformed mixtures act in a variety of ways, it is necessary to assess their toxicogenetic implications. On the other hand, most investigations on combined EDCs concentrated on analyzing acute hazardous effects rather than chronic effects, which is a major scientific study gap. The MoA of compounds varies depending on the composition, as do their impacts on reproductive functions. Predictive models might help develop quantified interspecies toxicogenetic differences of various EDCs mixtures. Proteomics, genomics, transcriptomics, and metabolomics should all be used together to study critical metabolic pathways used by the EDCs mixtures to impact upon the reproductive functions. The literature on transgenerational mixing effects of EDCs is scarce, thus additional study is necessary. Finally, despite the importance of prediction models (CI, IA, and CI), their shortcomings should be examined to ascertain accurate toxicological trends. The main flaw of the CA model is that modest effects might be found for different mixes. In contrast, the IA model implies that all mixed effects are fully independent of one another, which is impossible in reality. Some substances can change into metabolites and exert their effects. Thus, the CI model did not predict toxicity but rather showed obvious interactions among mixes at various degrees.

The link between EDC exposure and reproductive disorders is currently unclear. This is true for both general public and more vulnerable subpopulations. Most EDCs are ingested by humans. Inhalation and cutaneous exposure are irrelevant. For important developmental stages (e.g., pregnancy, infants, and kids), data on exposure levels are critically needed. Generally, fetal exposure is estimated from maternal levels, which may not correctly reflect exposure during important developmental phases. Cord blood and amniotic fluid samples may also not reflect important developmental phases. Conceptional exposure can be assessed using meconium. More information is needed on EDCs distribution, tissue level correlations, and excretory products to validate computer-generated exposure models. Many EDCs exist in complicated combinations of related compounds, causing measuring issues. We do not know much about the relative relevance of related substances in terms of hormonal action, much alone complicated combinations. Global prioritization of possible EDCs is required. Mechanisms for enhanced information exchange are also required to improve the comparability of national and regional EDCs monitoring programs.
